# Experimental Analysis of Seismic Damage to the Frame Structure–Site System Crossing a Reverse Fault

**DOI:** 10.3390/s25226866

**Published:** 2025-11-10

**Authors:** Jing Tian, Haonan Zhang, Shihang Qu, Jianyi Zhang, Hongjuan Chen, Zhijie Xu, Yijie Song, Ran Zhang

**Affiliations:** 1 School of Civil Engineering, Institute of Disaster Prevention, Sanhe 065201, China; tianjingle27@163.com; 2 Key Laboratory of Building Collapse Mechanism and Disaster Prevention, China Earthquake Administration, Sanhe 065201, China; 3 Key Laboratory of Earthquake Engineering and Engineering Vibration, Institute of Engineering Mechanics, China Earthquake Administration, Harbin 150080, China; 4 Longnan Mineral Exploration Institute, Gansu Nonferrous Metals Geological Exploration Bureau, Longnan 742500, China; 13643719328@163.com; 5 College of Geological Engineering, Institute of Disaster Prevention, Sanhe 065201, China; zhangjianyi@cidp.edu.cn (J.Z.); 15053170187@163.com (Z.X.); songyijie2000@163.com (Y.S.); 18156824913@163.com (R.Z.); 6 Institute of Geophysics, China Earthquake Administration, Beijing 100081, China; chenyu94@163.com

**Keywords:** reverse fault, cross-fault structure, isolated foundation, physical model test, dynamic response mechanism

## Abstract

Buildings crossing active faults often suffer severe damage due to fault dislocation during direct-type urban earthquakes. This study employs physical model tests to systematically investigate the dynamic response mechanisms of the integrated “surface rupture zone–overburden–foundation–superstructure” system subjected to bedrock dislocation. A testing apparatus capable of simulating reverse faults with adjustable dip angles (45° and 70°) was developed. Using both sand and clay as representative overburden materials, the experiments simulated the processes of surface rupture evolution, foundation deformation, and structural response under varying fault dislocation magnitudes. Results indicate that the fault rupture pattern is governed by the bedrock dislocation magnitude, soil type, and fault dip angle. The failure process can be categorized into three distinct stages: initial rupture, rupture propagation, and rupture penetration. The severity and progression of structural damage are primarily determined by the building’s location relative to the fault trace. Structures located entirely on the hanging wall exhibited tilting angles that remained below the specified code limit throughout the dislocation process, demonstrating behavior dominated by rigid-body translation. In contrast, buildings crossing the fault exceeded this limit even at low dislocation levels, developing significant tilt and strain concentration due to differential foundation settlement. The most severe damage occurred in high-angle dip sand sites, where the maximum structural tilt reached 5.5°. This research elucidates the phased evolution of seismic damage in straddle-fault structures, providing experimental evidence and theoretical support for the seismic design of buildings in near-fault regions. The principal theoretical and methodological contributions are (1) developing a systematic “fault–soil–structure” testing methodology that reveals the propagation of fault dislocation through the system; (2) clarifying the distinct failure mechanisms between straddle-fault and hanging-wall structures, providing a quantitative basis for targeted seismic design; and (3) quantifying the controlling influence of fault dip angle and soil type combinations on structural damage severity, identifying high-angle dip sand sites as the most critical scenario.

## 1. Introduction

Earthquakes in urban and adjacent areas can exacerbate building damage and ground disasters along causative faults. Particularly, “direct-type” earthquakes resulting from the sudden, rapid dislocation of active faults beneath cities can induce more severe structural damage.

**Figure 1:**
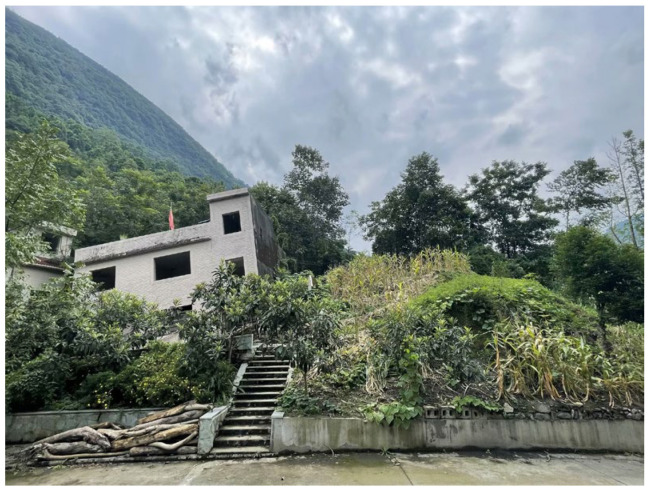
Damage to Typical Buildings in Shaba Village, Beichuan County.

Typical seismic damage cases demonstrate that a building’s position and distance relative to the surface rupture trace are critical factors influencing the severity of structural damage. For instance, Kelson [1] and Lin [2] presented a survey of building damage at Fengyuan Guangfu Middle School and its vicinity during the Chi-Chi earthquake. Structures located more than 50 m from the fault trace remained largely undamaged, whereas all buildings directly on the trace were destroyed or collapsed. R. Ulusay [3] investigated the damage to eight five-story apartments in Kullar village, located on a strike-slip fault with a normal fault component, during the Turkey İzmit earthquake. Two buildings directly intersected by the fault, along with six other hanging-wall structures situated 5–20 m from the surface rupture, completely collapsed. However, a building of the same structural type on the footwall, about 15 m from the surface rupture, survived the earthquake. In the Wenchuan earthquake, buildings in the Yingxiu town area, which is located on the hanging wall and intersected by the rupture trace, completely collapsed, while buildings on the footwall, approximately 20 m from the trace, remained intact [4,5,6,7]. Similar typical cases were also visually documented in Xiaoyudong Town [4,5,6] and Beichuan County [7] during the same earthquake (as shown in Figure 1). It is evident that the severity of damage to structures crossing faults is closely related to their distance from the surface fault rupture and their position relative to the hanging wall or footwall.

To mitigate these risks, numerous codes and regulations, both domestically and internationally [8,9,10,11,12,13], have established risk management strategies centered around setback distances. These provisions generally restrict or prohibit construction within zones extending tens to hundreds of meters on either side of the fault trace. However, from an engineering practice perspective, regulations based solely on setback distances suffer from significant limitations: On one hand, an approach focusing only on avoidance distances makes it difficult to develop risk control methods that are both economically feasible and scientifically sound. On the other hand, existing regulations fail to quantitatively elucidate the intrinsic relationships among different fault parameters, soil conditions, and structural responses, thus lacking the capability to provide a scientific and refined basis for seismic design.

To address the limitations of current codes, researchers have turned to model tests and numerical simulations to quantitatively reveal the fault–soil–structure interaction mechanisms. M Fadaee [14], through experiments and numerical simulations, confirmed that seismic damage to cross-fault buildings mainly depends on the differential rotation angles of foundations and the fault dislocation amount and that the fault rupture path is regulated by soil strength and foundation stiffness. Mehdi [15] quantified special seismic damage mechanisms of foundations (fault rupture, foundation voiding) through centrifuge tests, revealing that foundation embedment depth and stiffness can significantly improve foundation rotation and horizontal displacement, jointly controlling the building damage pattern. Mohammadreza [16] quantitatively analyzed the displacement, rotation, and moment responses of raft foundations to fault dislocation amount and relative fault position through 3D finite element simulations and qualitatively revealed key failure mechanisms such as fault rupture paths and soil–pile interface plastic yielding. Although these studies have successfully elucidated key mechanical mechanisms at the foundation level, their perspective is often confined to localized systems of “foundation–soil” or “foundation–fault”. The implications of this limitation are twofold: (1) they fail to adopt the integrated “surface rupture zone–overburden soil–subsoil–foundation–superstructure” system perspective, thereby lacking a systematic depiction of the complete process of fault dislocation’s progressive transmission and evolution through the system, and (2) a dedicated experimental comparison and analytical framework is lacking for the dynamic response differences and failure modes between the two most typical structural categories: “cross-fault buildings” and “hanging-wall buildings”.

Therefore, this study aims to address the limitations of existing research by conducting large-scale physical model tests to systematically investigate the seismic damage mechanisms of buildings and sites crossing reverse faults [17,18,19,20,21,22,23,24]. We constructed an experimental model representing the integrated “surface rupture zone–overburden–subsoil–foundation–superstructure” system to quantitatively analyze its failure process under bedrock dislocation. To achieve this objective, we designed and developed a testing apparatus capable of simulating reverse-fault dislocations with various dip angles. Using both sand and clay as representative overburden materials, we systematically investigated the effects of fault dislocation magnitude, dip angle, and soil type on surface rupture evolution, foundation deformation, and structural failure modes by comparatively analyzing the responses of straddle-fault and nearby fault structures. The core innovations of this work are as follows: (1) It transcends the foundation-centric perspective prevalent in previous studies by pioneering an integrated “fault–overburden–subsoil–foundation–superstructure” system approach, experimentally revealing the complete action chain and phased evolution of building damage induced by fault dislocation. (2) It clearly distinguishes and compares the dynamic responses and failure modes between “cross-fault buildings” and “hanging-wall buildings”. (3) Through comparative tests quantitatively elucidates the key mechanism whereby soil properties and fault geometry jointly govern structural failure patterns.

## 2. Engineering Background

Constructing a simplified model of the “surface rupture—overburden—foundation—superstructure” system and developing a rational testing scheme for its analysis represent a critical and urgent practical challenge in this field. UBC97 [8] stipulated that four conditions must be simultaneously met: (1) the building is located in seismic zone 4; (2) the fault is a seismogenic fault; (3) the fault is capable of generating earthquakes of Mw6.5 or greater; and (4) the fault distance is less than 15 km. Only then should the near-fault coefficient be considered. However, it did not provide specific values or calculation formulas for this coefficient. In 1999, the revised A-P guidelines by the California government [9] prohibited construction within 15 m on either side of a known fault trace. When the location of an active fault is uncertain, construction is forbidden within 150 m on each side, and for certain small active faults, the prohibition generally extends to 60–90 m on either side. In 2003, the Utah Department of Natural Resources [10] mandated that for certain faults, construction is not allowed within 75 m of the hanging wall trace and within 150 m of the footwall trace. For faults with uncertain locations, a 300 m prohibition was applied on both sides. That same year, the New Zealand Geotechnical Society [11] proposed that the potential hazard zone width for surface fault rupture is 10–50 m, and due to uncertainty, an additional 20 m buffer should be added beyond this zone. Furthermore, in 2006, the European Technical Committee [12] stated in a summary report that construction should be prohibited within 30 m on either side of a strike-slip fault trace. For dip-slip faults, a 30 m prohibition was applied to the hanging wall for both normal and reverse faults [13].

Developing a simulation test apparatus suitable for studying the seismic damage response of cross-fault structures and rationally designing its test scheme for analysis represents a pressing practical challenge in this field. Research by Deng [25] and Wang [26] showed that under the same magnitude conditions, a thin overburden or a shallow focal depth significantly increases the probability of surface rupture. Their findings indicated that when bedrock dislocation exceeds 1 m, it can lead to the formation of surface uneven deformation zones or rupture zones in overburden layers 20–50 m thick. In accordance with current building design codes for engineering sites [27,28,29,30], a specialized assessment of the engineering impact of potential seismogenic fault zones within a site is required under the following conditions: when the seismic fortification intensity is VII or IX degrees, and the thickness of the Quaternary overburden soil above buried bedrock faults is less than 60 m and 90 m, respectively. Therefore, by comprehensively considering the spatial scale of surface rupture effects, this paper selects a site with a 20 m thick overburden as the test simulation object.

Furthermore, achieving a realistic simulation of fault dislocation requires precise control of the dislocation rate. Analysis of seismic damage from strong earthquakes provides key parameters for this purpose. During the Wenchuan earthquake, a strike-slip thrust fault with a dip angle of approximately 33° was formed, featuring a co-seismic displacement of 6–9 m over a rupture process lasting about 90 s [31]. The reverse fault generated by the Chi-Chi earthquake caused a maximum surface vertical displacement of 4.4 m and a horizontal displacement of 10.1 m, with a rupture process duration of 102 s [32,33]. In summary, the fault dislocation rates in these two earthquakes were approximately 40–80 mm/s; therefore, based on a geometric similarity ratio of 1:20 for overburden thickness, this test selected a loading rate of 2 mm/s to simulate the fault dislocation process. Regarding the selection of the fault dip angle in the test, direct fault dislocation undoubtedly poses the most severe hazard to structures. Historical trenching statistics on fault dip angles [7,34,35,36,37,38,39,40] indicate that dip-slip faults with angles between 45° and 70° are relatively common. Considering that near-surface 20 m overburden commonly consists of Quaternary sandy soil and clay, this paper designs simulation tests for both normal and reverse-fault dislocations. Tests are conducted for the most unfavorable scenarios, with dip angles of 45° and 70°, under two site conditions (sandy soil and clay), to analyze the seismic damage mechanism of cross-fault structures.

Synthesizing the above analysis of building seismic damage under strong earthquake surface rupture conditions and drawing on relevant experimental devices developed by domestic and international scholars [14,15,16,41], the design and development of a physical simulation device for fault dislocation were completed. Concurrently, while fulfilling the requirements for building site tests, key components, including the data acquisition system, instrument layout, hydraulic loading platform, and control system, were systematically optimized.

## 3. Test Design

### 3.1. Model Device

**Figure 2:**
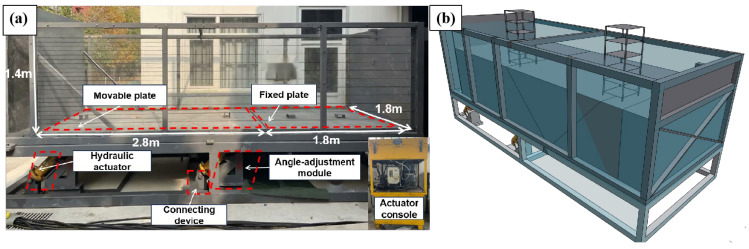
Experimental Device: (**a**) Model Box. (**b**) Experimental Layout.

The test apparatus for investigating the rupture behavior of large-scale overburden soil during strong earthquake bedrock fault dislocation comprises the following key components: a large-scale overburden soil box, a module for adjustable fault dip angle, four hydraulic actuators driving bedrock dislocation, a control platform for synchronizing actuator movement, connection support structures, and a reaction base (Figure 2). The soil box is designed with internal dimensions of 4.96 m in length, 1.85 m in width, and 1.40 m in height. Both the front and rear sides are fitted with high-strength, transparent acrylic plates, each 0.025 m thick, to facilitate observation of internal soil deformation. To enhance the overall stiffness, three vertical and one horizontal rigid ribs are installed on the exterior of the acrylic panels. The side panels of the soil box are constructed from 0.015 m thick steel plates and are further reinforced with welded X-shaped diagonal supports. The bottom of the soil box is designed as a split structure, consisting of an “L”-shaped movable bottom plate and a fixed bottom plate, used to simulate the active and passive plates in fault dislocation, respectively. When conducting reverse-fault dislocation tests, the adjustment module and hydraulic actuators corresponding to the desired angle are selected and hinged to the reaction base and movable bottom plate in a predetermined direction, with the movable bottom plate corresponding to the fault hanging wall.

The device is equipped with a PLC-based control system, integrating fault loading drive software, string potentiometer interfaces, hydraulic pipeline channels, and motor servo systems. During the test, the hydraulic actuators are connected to the console via oil pressure pipes, string potentiometers are arranged between the bedrock bottom plate and the base, and synchronized, precise loading and motion control of the actuators are achieved through dedicated software commands.

### 3.2. Materials

**Figure 3:**
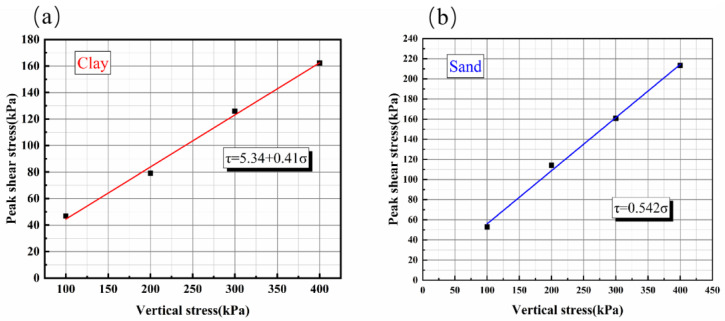
Shear strength curve of the soil: (**a**) Clay; (**b**) Sand.

This study aims to conduct a site test on structures near faults. Building upon extensive prior research on physical modeling of dip-slip faults [34,35,36] and incorporating seismic damage observations of buildings spanning reverse faults from the Chi-Chi and Wenchuan earthquakes [39,40], we designed experiments for sand and clay sites with dip angles of 45° and 70°, which are commonly observed in actual seismic damage. The objective was to simulate building damage across faults under realistic engineering conditions. Based on the seismic design code [30], which specifies an overburden thickness of 60–90 m for surface rupture, we determined a 1:30 similarity ratio after considering prototype building dimensions, model box capacity, boundary effects, laboratory conditions, and material strength constraints. This ratio was selected to establish a 100 mm thick soil layer in the model, representing a prototype overburden thickness of 20–30 m. The physical–mechanical similarity of the model is shown in Table 1. The physical and mechanical properties of the test soils are summarized in Table 2 and Figure 3.

This experiment uses a steel frame building structure model; building floors and structural columns are made of 65 Mn steel plates, and isolated foundation structures are made by proportionally mixing gypsum and other materials. The building model has 5 above-ground stories. The columns are steel plates with a height of 150 mm, width of 20 mm, and thickness of 2.5 mm. The floors are square steel plates with a side length of 300 mm and thickness of 1.5 mm, connected to the columns by welding. The materials for the isolated foundations are based on strength similarity theory. Orthogonal tests were conducted on test blocks cast with different water–cement ratios; the materials used were light gypsum powder, 425 Portland cement, and quartz coarse sand with a particle size of 0.5–1 mm. After standard concrete curing for 7 days, uniaxial compression tests were performed on each specimen to calculate the compressive strength of the test blocks.

**Figure 4:**
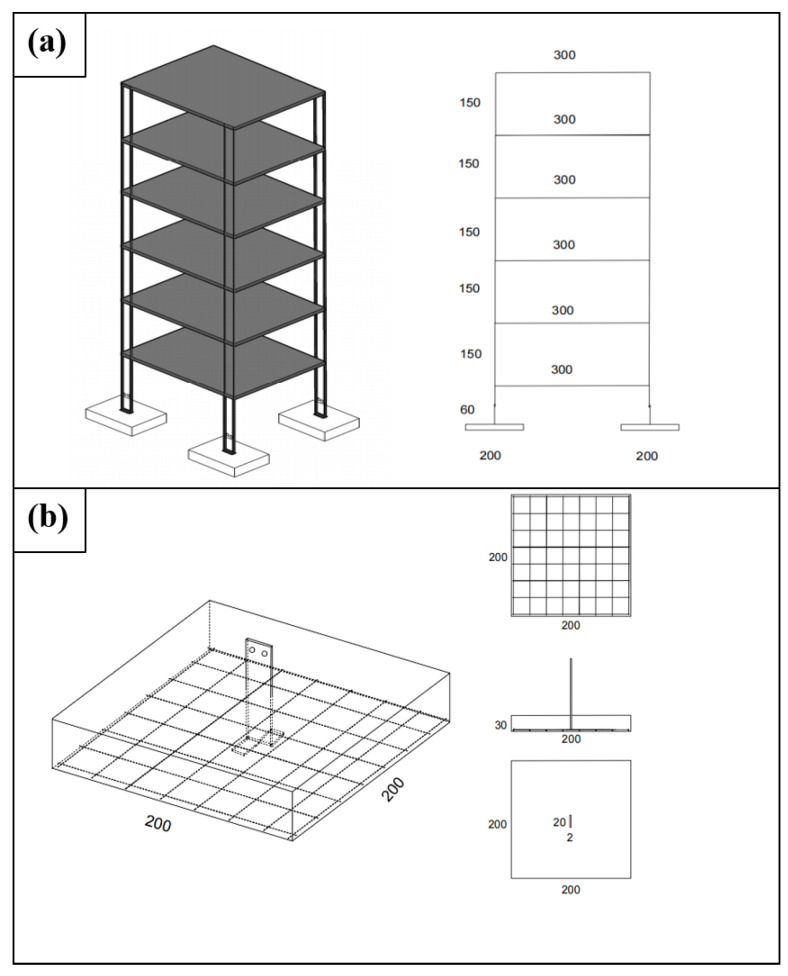
Building Model Parameters: (**a**) Design of Building Dimensions. (**b**) Design of Isolated Footings (unit: mm).

High-strength cast-in-place concrete was adopted for the column isolated footings to leverage the foundation’s stiffness in mitigating seismic damage for the cross-fault structure and to clearly capture its global failure pattern. According to the strength similarity theory, with a similarity ratio of 1:30 to simulate C30 concrete, the material ratio is cement/gypsum/sand/water = 3:12:30:12, and the compressive strength is 3.75 MPa. The foundation dimensions are 200 mm long, 200 mm wide, and 30 mm high, with reinforcement using Φ 2.4@25 mesh. The embedment depth of the isolated foundations is 90 mm. A schematic diagram of the fixed position between the isolated foundation and the column is shown in Figure 4.

### 3.3. Scheme

**Figure 5:**
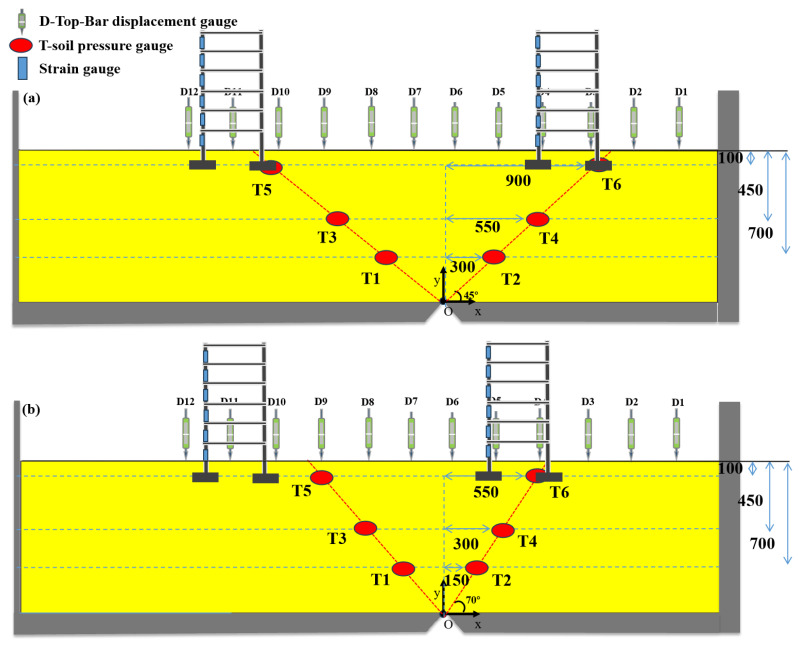
Instrumentation Layout Plan: (**a**) Test 1 and Test 2; (**b**) Test 3 (unit: mm).

Based on engineering background analysis, three sets of tests were arranged for cross-comparative analysis to investigate the site failure mechanism and building structural response in near-fault areas, focusing on the “surface rupture zone—overburden soil (foundation)—foundation—building structure” system while combining the damage to overlying soil caused by fault dislocation. The analysis aimed to explore the differences in site damage in near-fault areas and the damage differences under different fault dislocations in the same site. The instrument layout is designed as shown in Table 3 and Figure 5.

The monitoring scheme for soil response under reverse-fault dislocation was designed as follows: First, based on the development pattern of the main reverse-fault rupture zone identified in typical seismic damage cases and related experiments [38,42], earth pressure cells were arranged in a “V”-shaped array to directly capture the rupture path propagating from the bedrock to the surface. Additionally, displacement rods were installed on the ground surface to monitor differential uplift or settlement. These data, together with responses from strain sensors, are crucial for evaluating the deformation characteristics of the overlying structure. Table 4 lists the specific parameters of these sensors.

## 4. Analysis of Experiment Conclusions

Through large-scale physical model tests, this study systematically investigated the evolution of fault rupture and foundation failure modes, along with their impact on superstructure responses induced by bedrock dislocation under varying soil properties and fault dip angles. The test results indicate that the morphology of fault rupture and its damaging effect on structures are jointly governed by the amount of bedrock dislocation, differences in soil properties, and the fault dip angle. This chain of action can be summarized as a stagewise process: “bedrock dislocation drives fault rupture—local foundation instability—surface uneven deformation—structural translation and tilting failure”.

**Figure 6:**
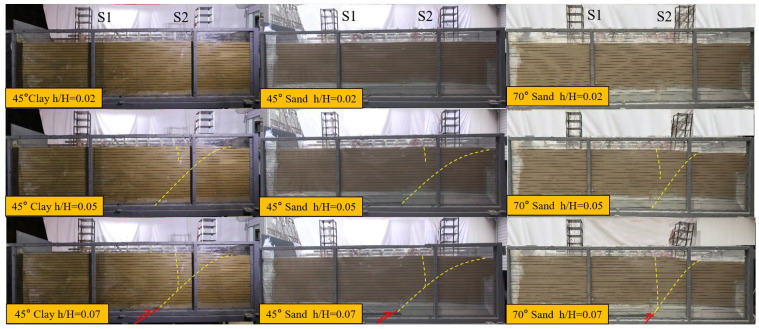
Macroscopic Fracture Profile.

From the model test cross-sectional diagrams (Figure 6), it can be observed that under bedrock dislocation, site damage under different soil conditions presents a process of gradual evolution from cracks to rupture zones. To facilitate the description of test conditions, the bedrock dislocation is expressed as h/H, where h is the bedrock dislocation and H is the overburden thickness (1 m):

1. The first stage is the initial rupture stage; under small dislocation amounts, surface rupture and deformation are not yet obvious, and soil pressure changes are significantly controlled by soil properties. In this stage, the structure only exhibits slight tilting and limited translation, with internal force changes mainly concentrated in local stress changes caused by initial soil rupture.

2. The second stage is the rupture development stage; when the dislocation amount gradually increases, the original rupture zone in sandy soil continues to expand, and a secondary rupture zone appears; in clay, a trace develops into a distinct scarp, accompanied by continuous growth of vertical displacement at the fault outcrop. In this stage, surface uneven deformation becomes obvious, the horizontal displacement of the structure under the three working conditions increases significantly, and the tilt degree of the house at the fault dislocation location increases markedly.

3. The third stage is the complete fault development stage; under large fault displacement, the rupture zone fully penetrates and forms a rupture plastic zone. Strong surface uneven deformation causes the structural response to shift from translation-dominated to tilt-dominated, with the most severe structural tilt occurring in Test 1.

### 4.1. Surface Deformation

**Figure 7:**
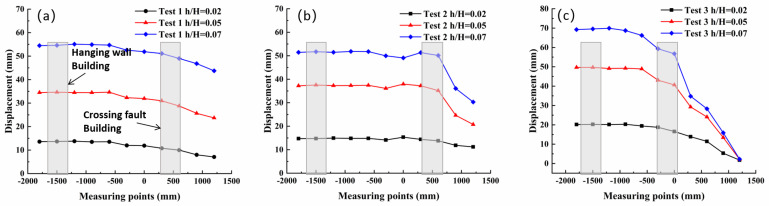
Surface Displacement: (**a**) Test 1; (**b**) Test 2; (**c**) Test 3.

Based on analysis of surface deformation measurements at the test site (Figure 7), as surface deformation intensifies, the hanging wall overburden basically maintains rigid-body motion following bedrock dislocation, which also ensures that the hanging-wall buildings do not exhibit significant deformation characteristics. However, large surface deformation scarps appear at the surface rupture locations; this surface deformation is more pronounced in high-dip-angle sandy soil sites, leading to severe tilting and foundation damage of the buildings thereon.

Overall analysis reveals that the fault dip angle and soil property differences directly affect the distribution and evolution mode of the surface rupture zone, thereby controlling the location and amount of uneven foundation deformation; the foundation failure mode determines the dominant form of structural damage: in the second stage, differential foundation deformation mainly causes structural translation, while in the third stage, large-scale uneven settlement and scarp deformation lead to increased structural tilting. In clay sites, due to the greater soil rigidity, surface deformation in the rupture zone is smaller, and structural tilt at the rupture location is less; in sandy soil sites, local deformation is larger, and tilting risk is greater, especially prominent at high dip angles.

### 4.2. Foundation Changes

**Figure 8:**
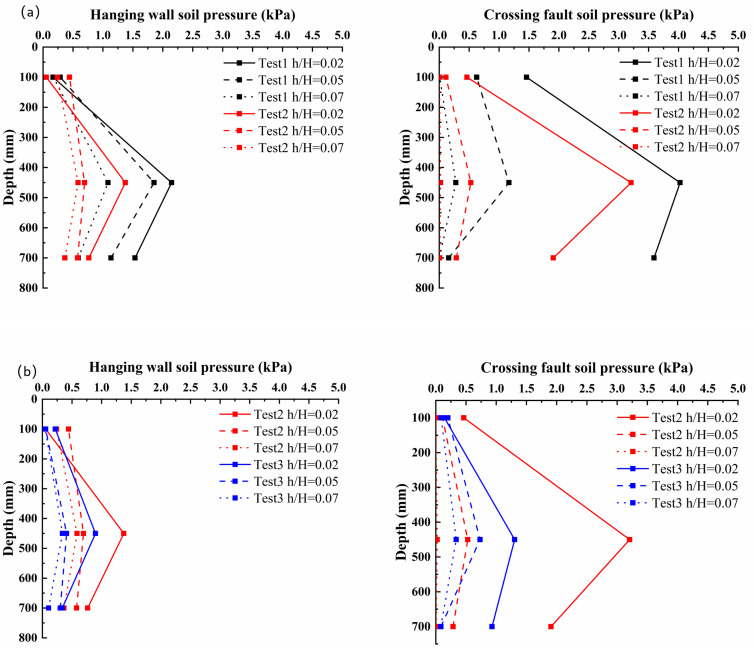
Variation in Earth Pressure with Depth in the Overburden Layer: (**a**) Comparison between Test 1 and Test 2. (**b**) Comparison between Test 2 and Test 3.

Soil type is a key factor determining the foundation response mode. The clay site (Test ①), due to its cohesion and higher stiffness, exhibited greater integrity. During the initial rupture stage, a significant and continuous increase in earth pressure was observed within the clay (as shown in Figure 8). This indicates that the soil resisted and transferred the stress generated by bedrock dislocation through its own deformation and the development of micro-fractures. The relatively slow development of the rupture zone allowed stress redistribution over a wider area, resulting in relatively minor and localized surface deformation. In contrast, the sand sites (Tests ② and ③), being loose and cohesionless, showed a sharp decline in earth pressure shortly after a brief increase once the shear rupture zone initially formed during the rupture propagation stage. This critical phenomenon signifies that the soil within the rupture zone had essentially failed completely and lost most of its bearing capacity. This leads to the formation of prominent surface scarps. Consequently, the extensive surface deformation observed in the sand sites is the direct result of the full development of the internal rupture zone.

On the other hand, the fault dip angle effectively regulates the rupture propagation path. The low-angle fault exhibited a longer rupture path, leading to greater energy dissipation during upward propagation. Consequently, rupture development was more gradual, and the plastic zone formed slowly and progressively. In contrast, the high-angle fault developed a nearly vertical rupture path. This allowed energy from the bedrock dislocation to be transmitted upwards more efficiently. This explains why the most abrupt pressure drop at the T3 sensor and the most prominent surface scarp were ultimately observed in Test ③. Considering the overall experimental results, the site conditions in Test ③, characterized by the combination of the “shortest energy transfer path” and “weaker soil stiffness”, collectively constituted the most critical scenario.

Therefore, overburden foundation failure is not an isolated phenomenon but rather part of the failure process of the entire “fault–overburden” system. The higher stiffness of clay sites, combined with the longer rupture path characteristic of low-angle faults, collectively leads to less severe damage. Conversely, the combination of lower stiffness in sand sites and the shorter rupture path of high-angle faults results in more severe damage characteristics.

### 4.3. Building Structure Damage

**Figure 9:**
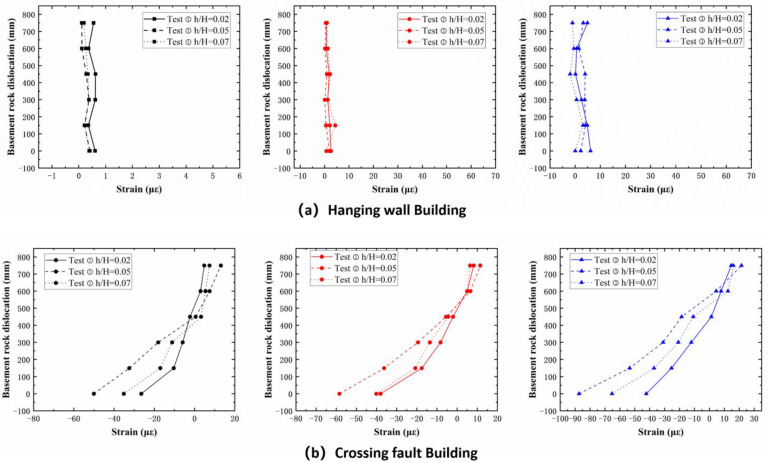
Structural Strain Response: (**a**) Hanging Wall. (**b**) Crossing Fault.

**Figure 10:**
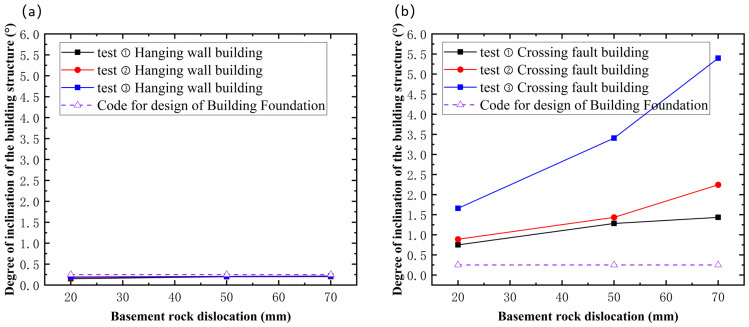
Structural Tilt: (**a**) Hanging-Wall Building. (**b**) Cross-Fault Building.

Based on the allowable values for building foundation deformation outlined in relevant codes and standards, and combined with an analysis of the tilt degree of the experimental building structures, the damage levels of the site building structures under fault dislocation with different dip angles were determined. Buildings located on the hanging wall essentially underwent rigid-body motion without significant tilting; the maximum tilt angle in Test 2 was only 0.25°. Judging from the strain results (as shown in Figure 9 and Figure 10), no significant damage occurred to the hanging-wall buildings.

Regarding standards for evaluating the severity of damage from building foundation settlement, McCalpin [43] established a correlation between ground rupture displacement and building damage severity, proposing that displacements below 0.1 m generally cause slight damage, while those between 0.1 and 0.3 m lead to serious but repairable structural harm. Displacements of 0.3–0.6 m are likely to cause irreparable damage, and values exceeding 0.6 m often result in collapse. Drawing on post-earthquake building damage analyses [44,45] and in alignment with the code for the design of building foundations [30], a foundation is defined as failed when the ratio of the differential settlement between two points to their distance exceeds 0.004 (0.25°).

Overall, the tilt angles of the hanging-wall structures remained well below the code-specified limit of 0.25° throughout the dislocation process (only 0.25° at maximum in Test ②). This observation, combined with strain data showing no significant variation, indicates that the hanging-wall structures underwent only rigid-body translation, remaining in an intact or slightly damaged state. In contrast, the damage to the cross-fault structure was significantly more severe and developed through distinct stages. During the initial minor damage stage, the structural tilt increased slowly, remaining at or near the 0.25° threshold, indicating elastic deformation and reversible damage. In the second stage, the tilt angle significantly exceeded the 0.25° limit and entered a period of rapid growth. For example, in Test ②, the tilt reached 2.25° by the end of this stage, nine times the code allowance. Concurrently, a sharp increase in flexural strain at the bottom story indicated the structure had entered the plastic deformation stage, suffering irreversible damage. In the third stage, the structural tilt angle peaked. In Test ③, the final tilt reached 5.5°, 22 times the code limit. This extreme differential settlement not only caused immense strain in the bottom-story columns but also raised the structure’s inflection point to the top story, signaling an imminent risk of global collapse.

Integrated analysis of the experimental data reveals that surface deformation and internal earth pressure changes clearly delineate the complete process of fault rupture propagation from bedrock to surface and its control over surface morphology: (1) During the initial rupture stage, surface displacement was minor. Correspondingly, earth pressure cells installed in the lower overburden were the first to respond. This indicates that the energy from bedrock dislocation had transferred into the overburden, inducing initial shear deformation at its base, although the rupture had not yet reached the surface. (2) In the rupture propagation stage, visible cracks emerged on the surface and gradually developed into scarps. The most critical evidence for this stage comes from the earth pressure data. Taking Test ③ as an example, the reading from sensor T3, located near the developing main rupture zone, increased initially and then dropped abruptly (Figure 8). This phenomenon was temporally consistent with the formation of the surface scarp at that location (Figure 7c). This indicated that when the shear rupture surface fully penetrated the soil, forming a continuous plastic slip zone, the soil within the rupture zone experienced stress release and a sharp reduction in bearing capacity. Therefore, the pressure drop recorded by T3 serves as direct evidence of the soil within the rupture zone transitioning to a plastic state and undergoing local instability. This internal instability caused the hanging-wall soil mass to slide relatively along this continuous weak plane, manifesting as significant scarp growth at the surface. (3) In the rupture penetration stage, the rupture zone was fully developed, and the internal soil had completely failed. At this point, earth pressure data no longer showed significant fluctuations. Subsequent dislocation increments were primarily converted into concentrated relative displacement along this rupture surface, resulting in the continued accumulation of surface scarp height.

Integrated analysis indicates that the maximum surface deformation observed in Test ③ (high-angle dip, sand) did not originate from the most intense internal stress response but rather resulted from the most fully developed rupture zone and the most extensive plastic zone, which allowed deformation to be most effectively concentrated and transmitted to the surface. In contrast, in the clay site and the low-angle dip site, although internal earth pressure changes occurred earlier and were more pronounced, their rupture zones developed more restrictedly and deformations were more dispersed, resulting in less prominent surface scarps compared to Test ③.

### 4.4. Hanging-Wall Effect

Experimental results indicate that the tilt angles of structures on the hanging wall remained below the code-specified limit of 0.25° in all three tests. However, in the clay site (Test ①), the earth pressure beneath the foundation of the hanging-wall structure (located approximately 2.5 m, prototype 75 m, from the surface rupture trace) showed a steady increase with the h/H ratio, indicating soil compression without local failure. Simultaneously, the structure’s tilt angle remained below 0.1°, and its horizontal displacement was consistent with that of the adjacent soil. This suggests that structures within a distance of 2.5 times the overburden thickness (2.5 H) from the fault trace in low-angle reverse-fault clay sites are relatively safe.

In contrast, in sand sites, although the hanging-wall structures at similar distances did not exhibit severe tilting themselves, the earth pressure data near their foundations revealed potential risks. In Test ③, the earth pressure cell located approximately 1.8 m from the main rupture zone showed significant fluctuations and even a declining trend. This indicates that intense fault dislocation had induced soil loosening within the hanging-wall sand mass, even at distances where the building’s foundation itself had not yet fully destabilized. Consequently, although the structure itself underwent rigid-body translation, its supporting soil had been disturbed, posing potential long-term stability concerns. Based on these observations, we recommend defining the safe hanging-wall effect zone in sand sites as within approximately 1.5 H from the fault trace.

Based on the response characteristics of hanging-wall structures and the overburden, this study proposes a preliminary “hanging-wall effect” relatively safe zone. However, the extent of this zone is strictly governed by soil properties and the fault dip angle.

## 5. Failure Mechanism

**Figure 11:**
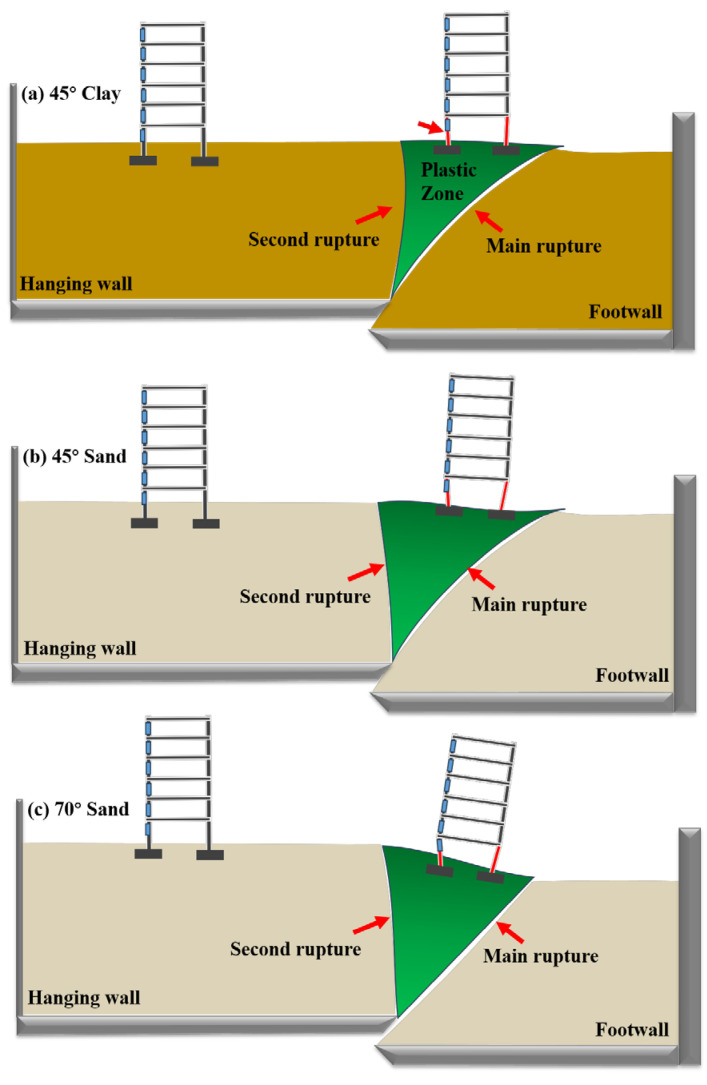
Failure Mechanism.

As shown in Figure 11, for the site with a frame structure on a low-angle reverse fault in cohesive soil overburden, the presence of the structure almost did not alter the propagation law of the seismic rupture through the free-field soil. The rupture still initiated at the boundary between the hanging wall and footwall of the bedrock, approximately along the fault dip angle line. A distinct rupture developed until it penetrated the entire overburden layer, forming a steep and wide main rupture zone characterized by uneven deformation at shallow depth. Analysis combining the macroscopic soil rupture pattern, surface uneven deformation, and earth pressure changes indicates that the damage within the main surface rupture zone was more severe in the cohesive soil overburden site containing the frame structure. This is attributed to the significant change in earth pressure observed in the middle part of the overburden within the main rupture zone.

Similarly, for the site with a frame structure on a low-angle reverse fault in sandy soil overburden, the structure scarcely changed the propagation law of the seismic rupture through the free-field soil. A main rupture zone appeared in the shallow soil layer along the fault dip line, thrusting towards the footwall. Additionally, a depression (a “\” direction depression and “/” direction uneven deformation) occurred within a certain area of the shallow soil on the hanging wall side of the main rupture zone. Analysis integrating the macroscopic soil rupture morphology, surface uneven deformation, and earth pressure variations reveals that for the sandy soil overburden site with a frame structure, severe damage occurred not only within the main surface rupture zone but also in the depressed area on the hanging wall side. The reason is that significant earth pressure changes were recorded in the overburden at these two locations.

On the other hand, the superstructure is not merely a passive recipient; it exerts a non-negligible back-action on the foundation and subsoil through its stiffness and self-weight. This interaction manifests specifically in the following two aspects: (a) Restriction of Free Deformation in the Hanging-Wall Soil: The self-weight of a structure located on the hanging wall increases the confining pressure on the soil, thereby enhancing its shear strength. This, to some extent, suppresses the free development of secondary ruptures within the hanging-wall zone, forcing deformation energy to be released more concentratedly along the main rupture zone. Consequently, this intensifies the “rigid-body translation” effect observed in buildings on the hanging wall. (b) Taking a building crossing the fault as an example, the significant flexural strain at its bottom story indicates that substantial additional bending moments are generated at the column–foundation connections. This bending moment acts in reverse on the foundation and is transferred to the subsoil, causing the soil beneath the foundation to enter a plastic state earlier and more severely, thereby accelerating local instability of the foundation.

The systematic experimental model of the “fault–overburden–foundation–superstructure” system developed in this study, along with its findings, overcomes the limitations of previous research methods that focused on isolated subsystems like “foundation–soil” or “foundation–fault”. It provides a scientific basis and key technical support for transitioning from “qualitative avoidance” to “quantitative design” in the seismic design and risk management of buildings in near-fault regions. Its core engineering application value is demonstrated in the following aspects:

1. Based on specific fault parameters and detailed geotechnical investigation results, the strictest avoidance criteria should be implemented for confirmed worst-case scenarios involving high-angle faults traversing sand sites. For low-angle faults traversing clay sites, however, setback distances could be appropriately optimized following the implementation of effective mitigation measures.

2. The study clearly distinguishes the failure mechanisms between “straddle-fault buildings” and “hanging-wall buildings,” establishing a basis for developing differentiated design strategies. For hanging-wall buildings that cannot be avoided, the design should prioritize structural integrity to safely dissipate energy from rigid-body translation while also ensuring subsoil stability to prevent structural failure triggered by local soil instability. For buildings that must straddle the fault, the design focus should be on accommodating large deformations, incorporating special structural details at predicted surface rupture and fault locations that allow for substantial relative rotation and displacement, thereby effectively releasing fault dislocation energy and protecting the main structural system.

In summary, this study advances fault-crossing seismic damage analysis from a problem primarily concerning setback distances to one that enables analysis and facilitates enhanced building fault rupture resistance during the initial design phase. It establishes a solid theoretical foundation and provides a clear practical pathway for implementing performance-based seismic design in near-fault regions.

## 6. Conclusions

This paper systematically investigates the dynamic response mechanism of the “surface rupture zone–overburden–foundation/substructure–superstructure” system under bedrock dislocation through a series of physical model tests.

The experiment utilized a reverse-fault simulation device with adjustable dip angles (45° and 70°) and selected two typical overburden soils, sand and clay, to simulate the processes of surface rupture evolution, foundation deformation, and structure response under increasing fault dislocation amounts. The main conclusions are as follows:

1. The evolution of fault rupture demonstrates distinct stage-specific characteristics. Fault rupture evolution exhibits distinct stage characteristics. The tests clearly reveal that the overburden failure process induced by fault dislocation can be divided into three stages: the initial rupture stage, the rupture development stage, and the rupture penetration stage. In the initial stage, minor bedrock dislocations result in negligible surface deformation, the formation of initial soil cracks, and a weak structural response. As dislocations increase, the rupture development stage is characterized by the propagation of the main rupture zone and secondary cracks, significantly aggravated uneven surface deformation, and a notable increase in structural horizontal displacement. Finally, during the rupture penetration stage, large dislocations cause the rupture zone to fully penetrate the overburden, forming a widespread plastic zone and distinct surface scarps, while the structural response transitions from being predominantly translation-dominated to tilt-dominated.

2. Soil properties and fault dip angle jointly control the failure mode of structures crossing the fault. For sandy sites, due to the loose soil and concentrated deformation, pronounced surface scarps develop. Particularly under the high-dip-angle condition, the building tilt was the most severe, with differential foundation settlement reaching 20 mm and a strong structural strain response. For clay sites, the greater soil stiffness leads to a more concentrated rupture zone development and relatively smaller surface deformation. The building tilt was lower, but significant bending strain at the bottom level was still evident. A higher fault dip angle results in a steeper rupture zone, more pronounced fracturing in the overlying soil, and consequently a stronger tilting effect on buildings.

3. The building’s location relative to the fault determines its damage stage and severity. The tilt of hanging-wall structures remained below the code limit of 0.25° throughout the dislocation process, maintaining an essentially intact state. In contrast, the structure crossing the fault exceeded this limit at a dislocation of approximately 30 mm (prototype 0.6 m). Its tilt angle increased sharply with further dislocation, reaching up to 22 times the code limit, resulting in destructive damage.

4. The foundation failure mode dictates the superstructure response. Minor uneven surface deformation induces structural translation, whereas significant soil faulting and plastic zone development lead to aggravated tilting. The test results indicate that foundation stiffness plays a crucial role in modulating deformation transfer. Isolated foundations are prone to rotation and differential settlement under fault dislocation, subsequently causing bending and shear failure in the superstructure. Future experimental designs could explore the influence of foundation type on the seismic damage characteristics of buildings crossing faults.

5. To highlight the processes of surface rupture evolution, foundation deformation, and structural response under varying fault dislocation magnitudes, this study employed a highly simplified frame model with an isolated spread footing. However, a key limitation is that this simplified structural and foundation system may not fully capture the complex response characteristics of actual buildings: (a) The isolated footing used in the test exhibits minimal capacity for coordinated action under fault dislocation. This directly transfers differential settlement and foundation rotation to the superstructure, likely amplifying the observed structural tilting and concentration of strain in the bottom story. (b) The simplified stiffness and integrity of the superstructure make it difficult to replicate the complex collapse modes that real structures undergo under large deformations. Therefore, future research should aim to develop larger-scale foundation–structure system models that more closely represent reality, specifically by conducting comparative studies on the performance of isolated footings, raft foundations, and pile foundations under identical fault dislocation conditions. This will help clarify the regulatory effect of different foundation types on seismic damage patterns, thereby providing more precise guidance for engineering design.

6. A limitation of this study is the lack of replicate tests for verification. In physical modeling, random factors such as soil compaction density, initial stress state, and minor construction discrepancies may introduce contingent variations in the surface rupture path, its development timing, and the structural response. The absence of replicate tests makes it difficult to quantify the potential range of this stochastic scatter on the quantitative results. This study established multiple comparative scenarios and observed consistent, physically explainable stagewise failure processes across different conditions. Nevertheless, conducting replicate tests on key scenarios in future work is essential to verify result reproducibility and better quantify uncertainty.

This study, through experimentation, verifies the evolution path and key influencing factors of seismic damage in buildings crossing faults, providing a significant scientific basis for future seismic design and risk assessment of buildings in near-fault regions.

## 7. Patents

[1]. Zhang Jianyi, Guo Xun, Bo Jingshan, Wang Qiang, Zhang Haonan. Simulation Device and Simulation Method for Building Avoidance Distance Adjacent to Surface Rupture Zones of Strong Earthquakes. China, CN111473934A.
[2]. Zhang Jianyi, Wang Qiang, Zhang Zhizhou, Wang Tuo. An Experimental Device for Simulating Surface Rupture Deformation of Reverse Faults under Earthquakes. China, CN110411821A.
[3]. Zhang Jianyi, Guo Xun, Sun Zhiguo, Wang Qiang. Simulation Device and Simulation Method for Bridge Damage under Near-Fault Ground Motion. China, CN111521364A.
[4]. Zhang Jianyi, Xu Zhijie, Li Zhongheng, Zhang Haonan. Test Simulation Device and Method for Building Structure Fracture Resistance under Cross-Fault Action. China, CN115541156A.

## Figures and Tables

**Table 1:** Scaling factors in the model test.

Number	Physical Quantity	Soil	Structures
1	Length (L)	$C_{L}=30$	$C_{L}=30$
2	Density (ρ)	$C_{\rho }=1$	$C_{\rho }=1$
3	Gravity (g)	$C_{g}=1$	$C_{g}=1$
4	Soi pressure (τ)	$C_{\tau }=C_{L}C_{\rho }C_{g}=30$	
5	Top-bar displacement (L)	$D_{L}=30$	
6	Elastic modulus (E)	D_E_ = 30	
7	Stresses (σ)		$C_{\sigma }=C_{L}C_{\rho }C_{g}=30$
8	Strains (ϵ)		$C_{\epsilon }=1$

**Table 2:** Scaling Factors for Physical Modeling.

Soil Type	Maximum Dry Density *ρ_d,max_* (g/cm^3^)	In Situ Dry Density *ρ_d_* (g/cm^3^)	Water Content *w* (%)	Cohesion c (kPa)	Internal Friction Angle φ (°)
Soil	1.673	1.588	5.4	0	28
Clay	1.870	1.728	25.5	5.34	21

**Table 3:** Summary of Test Conditions.

Test Case	Test ①	Test ②	Test ③
Overburden Thickness	1000 mm	1000 mm	1000 mm
Soil Type	Clay	Sand	Sand
Site Condition	Site with Frame Structure	Site with Frame Structure	Site with Frame Structure
Fault Type	Reverse Fault	Reverse Fault	Reverse Fault
Dip Angle	45°	45°	70°
Total Displacement	100 mm	100 mm	100 mm

**Table 4:** Sensor parameters.

Sensor Category	Sensor Model	The Parameters
Top-bar	YHD-200	1. Range: ±100 mm; 2. Bridging mode: full bridge or half bridge; 3. Sensitivity: 1.005.
Earth pressure cell	ESP-II	1. Range: 50 kPa/100 kPa; 2. Measurement type: voltage.
Strain sensor	BX120-5AA	1. Size: 5 mm × 3 mm; 2. Sensitivity: 2.08 ± 1%.

## Data Availability

The data that support the findings of this study are available from the corresponding author upon reasonable request.
